# Supplementary material for: Identification, analysis and prediction of valid and false information related to vaccines from Romanian tweets
Source: Front Public Health. 2024 Feb 1;12:1330801. doi: 10.3389/fpubh.2024.1330801 (PMC10867260; doi:10.3389/fpubh.2024.1330801)
Supplement: Supplementary file 1 [file Table_1.docx]

**Supplementary Table 1. Detailed example of implementation of SVM algorithm on 9 extra tweets from the external dataset**

| **Reformulated tweet content** | **Predicted probability (true)** | **Predicted probability (neutral)** | **Predicted probability (fake)** | **Predicted class** | **Annotated class/**  **Observation** |
| --- | --- | --- | --- | --- | --- |
| **Tweet D:**  Even though you are vaccinated, you still could catch the virus. There are persons who developed severe adverse reactions related to the (Covid) vaccine, while others developed none. We are all different! | 20.59% | 34.65% | 44.76% | Class 2 (fake) – erroneous prediction | Class 0 (true) |
| **Tweet E:** The first batch of Johnson vaccine has arrived in Romania. | 63.94% | 16.37% | 19.69% | Class 0 (true) | Class 0 (true) |
| **Tweet F:** USA has suspended the vaccination with Johsnon & Johnson. The company delays the vaccine launch in Europe. | 70.14% | 8.32% | 21.54% | Class 0 (true) | Class 0 (true) |
| **Tweet G:** There will be lots of controversies regarding Covid vaccination. In order to be able to talk more, I’m waiting for the law project. | 16.62% | 45.74% | 37.64% | Class 1 (neutral) | Class 1 (neutral) |
| **Tweet H:** The golden vaccines. | 4.13% | 25.23% | 70.64% | Class 2 (fake) – erroneous prediction | Class 1 (neutral) |
| **Tweet I:**  That he/she is getting the vaccine: | 0.51% | 96.3% | 3.19% | Class 1 (neutral) | Class 1 (neutral)  Observation: many tweets marked as neutral were short and/or lacked context. |
| **Tweet J:**  Researchers warn that vaccinating people who already had Covid-19 might cause very severe adverse reactions or even death! | 33.29% | 12.23% | 54.48% | Class 2 (fake) | Class 2 (fake)  Observation: not entirely fake, but misleading content |
| **Tweet K:** I agree with the mask. But with the vaccine..It’s debatable. | 13.76% | 28.89% | 57.35% | Class 2 (fake) | Class 2 (fake)  Observation: not fake, but misleading content. |
| **Tweet L:** We are study material…”The raise in death numbers is higher among those which received two vaccine doses” | 18.88% | 19.29% | 61.83% | Class 2 (fake) | Class 2 (fake) |
